# Threat-related AI anxiety and engagement in live-streamed AI courses: a moderated serial mediation model of extrinsic motivation and teacher support

**DOI:** 10.3389/fpsyg.2026.1831785

**Published:** 2026-04-29

**Authors:** Ming Lei, Yue Zou, Xin Wang

**Affiliations:** 1 School of Media and Communication, Yangtze Normal University, Chongqing, China; 2 School of Communication, Fujian Normal University, Fuzhou, Fujian, China; 3 School of Journalism and Communication, Chongqing University, Chongqing, China

**Keywords:** AI anxiety, extrinsic motivation, learning engagement, live streaming, self-efficacy, teacher support

## Abstract

**Background:**

As artificial intelligence becomes increasingly embedded in education and work, live-streamed AI courses have become a common format for timely, interactive, and practice-oriented instruction. At the same time, AI-related uncertainty has intensified learners’ anxiety. This study examined how threat-related AI anxiety is associated with engagement in live-streamed AI courses through extrinsic motivation for AI learning and perceived continuous teacher support, and whether this process varies by AI learning self-efficacy.

**Methods:**

A questionnaire survey was conducted among 487 adult learners in China who had participated in at least one live-streamed or synchronous AI-related course within the previous 6 months. Data were analyzed using PROCESS Model 87 with 5,000 bootstrap samples.

**Results:**

Threat-related AI anxiety was positively associated with extrinsic motivation for AI learning. Extrinsic motivation was positively associated with both perceived continuous teacher support and engagement, and perceived continuous teacher support was positively associated with engagement. The serial indirect effect through extrinsic motivation and perceived continuous teacher support was significant. AI learning self-efficacy significantly weakened the association between perceived continuous teacher support and engagement, such that learners with lower self-efficacy depended more heavily on teacher support for their engagement.

**Conclusion:**

Engagement in live-streamed AI courses is associated with a linked process involving emotional pressure, motivational activation, and instructional support. The study extends research on AI anxiety from motivation to engagement, identifies continuous teacher support as a key mechanism in live-streamed AI learning, and highlights the importance of accessible and supportive course design, especially for learners with lower AI learning self-efficacy.

## 1 Introduction

The rapid integration of artificial intelligence (AI) into educational systems has significantly influence how learners interact with knowledge and how institutions deliver instruction (Kaplan and Haenlein, 2019). AI-powered tools, spanning adaptive instructional platforms to data-informed learning support systems, are increasingly influencing what learners are expected to know and how they are expected to learn (Chiu, 2023). In this landscape, AI-related education constitutes a form of applied education—one that demands not only the acquisition of conceptual knowledge but also the development of practical competencies for navigating AI tools, understanding algorithmic processes, and maintaining relevance in a technology-driven society. Recognizing this, educational institutions worldwide have accelerated the incorporation of AI into curricula, with the number of courses utilizing AI increasing by over 100% in recent years (Zhang et al., 2021). Because AI technologies evolve at an unprecedented pace, live-streamed AI courses have emerged as a dominant and necessary format for delivering timely, interactive, and practice-oriented instruction. However, the immense urgency for adult learners to keep pace with these rapid, technology-driven educational changes generates intense psychological pressure. The psychological dimensions governing whether such rapid changes can yield effective, equitable, and sustainable learning outcomes remain insufficiently understood.

Among these psychological dimensions, AI anxiety has emerged as a particularly salient concern. AI anxiety refers to a general affective response of fear or unease arising from the perceived demands of interacting with, learning about, or keeping pace with AI technologies (Wang and Wang, 2022). Unlike traditional computer anxiety, AI anxiety is intensified by the autonomous decision-making capacity, self-evolving nature, and ethical opacity of AI systems (Li and Huang, 2020; Johnson and Verdicchio, 2017). A growing body of empirical evidence confirms that specific dimensions of AI anxiety, such as AI learning anxiety, can negatively affect students’ intrinsic motivation and behavioral intentions toward AI-related learning (Wu and Li, 2025; Wang et al., 2024). Critically, however, anxiety does not uniformly inhibit learning. The Control-Value Theory of Achievement Emotions (CVTAE; Pekrun, 2006) posits that achievement-related emotions, including anxiety, may vary in their functional consequences depending on individuals’ appraisals and contextual conditions.

Recent empirical work has demonstrated that certain threat-related dimensions of AI anxiety can serve a facilitating function. Wang et al. (2024) found that while AI learning anxiety is detrimental, AI job replacement anxiety acts as a facilitating factor that is positively associated with students’ extrinsic motivation to learn AI in order to maintain future employability. Furthermore, Teke (2025) revealed through latent profile analysis that learners categorized with “high AI risk awareness” actually demonstrated the highest levels of motivated learning behavior, as they frame technological threats as opportunities for personal growth rather than insurmountable obstacles. This dual-function perspective opens an important theoretical space: rather than treating AI anxiety solely as a barrier, researchers should examine the mechanisms and boundary conditions—such as AI learning self-efficacy, which significantly moderates how motivational states are associated with actual learning intentions (Wang et al., 2024; Wu and Li, 2025)—under which threat-related anxiety is associated with adaptive educational behavior.

Despite this emerging recognition, three critical gaps persist in the current literature. First, while prior studies have documented that specific dimensions of AI anxiety—namely, AI job replacement anxiety—are positively associated with extrinsic learning motivation (Wang et al., 2024), the downstream consequences of this motivational activation remain underexplored. Specifically, it is unclear how externally driven motivation is associated with learners’ perceptions of instructional environments and, subsequently, their actual engagement in AI learning. According to self-determination theory (SDT), learners driven by external pressures, such as the need to maintain competitiveness, actively seek out facilitating conditions to achieve their instrumental goals (Li et al., 2025). However, existing models typically stop at the motivation stage, without tracing how this externally driven urgency is associated with learners’ orientation toward continuous teacher support, which may in turn be linked to their behavioral, cognitive, and emotional engagement. This represents a crucial process-level gap.

Second, the role of instructional context, particularly continuous teacher support in live-streamed AI courses, has received little attention. Live-streamed courses represent an increasingly prominent modality for AI education, offering real-time instruction, sustained teacher availability, and iterative content delivery. These features are especially relevant for applied AI learning, where content evolves rapidly and learners face ongoing uncertainty about what knowledge is current and applicable. As highlighted by systematic reviews on online learning, learners frequently experience transactional distance and isolation in digital environments; thus, instructor support and teaching presence are essential for reducing anxiety and sustaining engagement (Hu and Xiao, 2025). Furthermore, perceived teacher support is instrumental in fulfilling students’ psychological needs for competence and relatedness (Shao et al., 2025). Yet, the mechanisms through which learners’ externally driven motivational states interact with perceived teacher support in this specific live-streamed format remain unspecified.

Third, although self-efficacy has been widely recognized as a determinant of learning engagement (Bandura, 1977; Shao et al., 2025), its moderating function on the link between perceived teacher support and engagement has not been fully tested in dynamic AI learning contexts. While recent studies have modeled academic self-efficacy as a mediator (Shao et al., 2025), empirical evidence also confirms that learning self-efficacy significantly moderates the association between motivational states and actual learning intentions in AI environments (Wang et al., 2024). In remote and digital learning environments, learners’ self-efficacy strongly influences their capacity to manage strategic learning and cope with psychological challenges (Teng et al., 2023). In the context of live-streamed courses, self-efficacy may condition the extent to which perceived teacher support is associated with engagement. Specifically, learners with lower self-efficacy tend to perceive remote interactive activities as more challenging and are less alert in seeking opportunities to engage with instructors (Lin et al., 2017; Teng et al., 2023). Even with continuous external support, learners with low AI self-efficacy may experience heightened self-doubt and withdraw effort when confronting complex AI algorithms (Teng et al., 2023; Wang et al., 2024). Consequently, continuous teacher support may function as a vital compensatory mechanism specifically for those lacking internal confidence. Thus, identifying how AI learning self-efficacy conditions the link from perceived teacher support to engagement is necessary to determine for whom instructional support is most consequential.

To address these gaps, the present study employs a quantitative, cross-sectional survey design. The empirical data were collected from a purposive sample of 487 adult learners in China who had actively participated in at least one live-streamed or synchronous AI-related course within the previous 6 months. By using a comprehensive questionnaire to capture learners’ real-world experiences, this study examines the sequential psychological and contextual processes associated with online learning participation. Through conditional process analysis, the research tests both the underlying mechanism linking threat-related AI anxiety to engagement and the role of AI learning self-efficacy in conditioning this process.

This research offers three primary contributions. First, it extends the literature on technology-induced emotion by specifying the serial process through which a negative emotional state may be linked to educational participation, rather than merely documenting its motivational correlates. Second, it highlights the critical role of instructional context by positioning continuous teacher support as an important mechanism linking motivational pressure to active engagement in live-streamed environments. This helps clarify how supportive instructional conditions may channel technological threat into learner agency (Wu and Li, 2025). Third, it refines current understandings of individual differences by examining AI learning self-efficacy as a boundary condition on the link from perceived continuous teacher support to engagement, clarifying for whom instructional support is most closely associated with active participation.

The remainder of this paper is structured as follows. Section 2 reviews the existing literature and formulates the research hypotheses. Section 3 outlines the research methodology, detailing the participant sample, data collection procedures, and measurement instruments. Section 4 presents the results of the statistical analyses and hypothesis testing. Section 5 discusses the theoretical contributions and practical implications of the findings, while also acknowledging the study’s limitations and suggesting directions for future research. Finally, Section 6 provides a brief conclusion.

## 2 Hypotheses development

### 2.1 Threat-related AI anxiety

AI anxiety, rooted in the broader construct of computer anxiety, refers to an individual’s affective response of fear, apprehension, or agitation that inhibits interaction with AI technologies (Wang and Wang, 2022; Johnson and Verdicchio, 2017). Whereas computer anxiety generally concerns unease about computer use (Parasuraman and Igbaria, 1990), AI anxiety is distinct in several respects. AI systems can make autonomous decisions, operate with limited transparency, and raise ethical concerns that extend beyond those associated with conventional computing (Li and Huang, 2020). These characteristics amplify the psychological impact of AI on learners, producing a form of anxiety that extends beyond technological unfamiliarity to encompass occupational, ethical, and existential concerns.

Wang and Wang (2022) identified four constituent dimensions of AI anxiety: learning anxiety, AI configuration anxiety, job replacement anxiety, and sociotechnical blindness. This multidimensional operationalization has been adopted and validated in subsequent research (Teke, 2025; Wang et al., 2024; Wu and Li, 2025). In applied AI education, learners may encounter all of these dimensions to varying degrees, as they face the cognitive demands of learning AI, the uncertainty associated with increasingly autonomous systems, concerns about occupational displacement, and broader apprehension regarding the social consequences of AI. As Wu and Li (2025) noted in their systematic review, AI anxiety in educational contexts is not merely fear of the technology itself, but also reflects concern about future competence, professional identity, and the ability to remain relevant in a rapidly changing environment.

A critical insight from recent research is that AI anxiety does not function uniformly as a debilitating force. Drawing on the Control-Value Theory of Achievement Emotions (CVTAE; Pekrun, 2006), anxiety can be understood as a negative activating emotion that may energize behavior under certain appraisal conditions (Pekrun et al., 2002). Likewise, the distinction between facilitating and debilitating anxiety suggests that anxiety can enhance adaptive action when it mobilizes resources toward reducing the discrepancy between one’s current and desired state (Alpert and Haber, 1960; Piniel and Csizér, 2013). In the context of AI education, this distinction is especially important because the very features that make AI anxiety distressing—rapid technological change, competitive pressure, and occupational uncertainty—may also compel learners to take action.

Recent evidence suggests that this facilitating function is especially evident in the threat-related dimensions of AI anxiety. Teke (2025) showed that pre-service teachers with high AI risk-awareness exhibited the highest levels of motivated learning behavior. Similarly, Wang et al. (2024) found that although AI learning anxiety negatively affected intrinsic motivation, AI job replacement anxiety positively predicted extrinsic motivation. These findings suggest that not all dimensions of AI anxiety operate in the same way. Rather, the dimensions most closely tied to perceived threat—particularly job replacement anxiety and sociotechnical blindness—may be especially likely to activate externally driven motivation to learn AI. Accordingly, the present study focuses on threat-related AI anxiety as the focal antecedent in the proposed model.

### 2.2 Extrinsic motivation

Self-determination theory (SDT; Deci and Ryan, 2000) provides a useful framework for understanding the quality of motivation that underlies learning behavior. Within SDT, extrinsic motivation refers to engagement in an activity for instrumental reasons, such as obtaining valued outcomes, meeting social expectations, or avoiding negative consequences, rather than for the inherent satisfaction of the activity itself (Teo et al., 1999; Ryan and Deci, 2020; Li et al., 2025). In AI learning contexts, extrinsic motivation is particularly salient because AI-related knowledge and skills are frequently framed as urgent professional necessities, and learners may approach AI education not only out of interest, but also because external pressures make such learning feel necessary (Wang et al., 2024).

The relationship between threat-related AI anxiety and extrinsic motivation can be understood through the activating function of negative emotions. When learners experience threat-related concerns, such as fear of job displacement, loss of future competitiveness, or broader sociotechnical risks, they are more likely to construe AI learning as something they ought to pursue in order to manage external demands and avoid negative outcomes. This appraisal pattern aligns with the external and introjected forms of regulation described by SDT. As Li et al. (2025) argued, learners driven by external and introjected regulation in AI contexts engage in learning to maintain self-esteem, avoid the guilt of falling behind, and cope with social and professional pressure. In such cases, motivation is shaped less by enjoyment of the learning activity itself than by its perceived instrumental necessity.

Empirical evidence supports this reasoning. Wang et al. (2024) demonstrated that while AI learning anxiety could undermine more autonomous forms of motivation, AI job replacement anxiety positively predicted extrinsic motivation among university students. This finding suggests that threat-related components of AI anxiety may activate externally regulated motivation as a coping response to perceived employability risks. Teke (2025) likewise reported that learners with the highest levels of AI risk-awareness displayed the strongest motivated learning behavior, a pattern consistent with the CVTAE proposition that anxiety, when paired with high subjective value, can energize approach-oriented action (Pekrun, 2006). Taken together, these findings suggest that threat-related AI anxiety may strengthen learners’ externally driven reasons for acquiring AI-related knowledge and skills.

Accordingly, the present study proposes the following hypothesis:

*H1*: Threat-related AI anxiety is positively associated with extrinsic motivation for AI learning.

### 2.3 Perceived continuous teacher support

Motivation not only energizes behavior but also shapes what learners attend to, value, and seek within a learning environment. According to self-determination theory, individuals with externally regulated motivation are especially oriented toward environmental features that help them achieve instrumental goals, such as clear guidance, performance-relevant feedback, and accessible expertise (Ryan and Deci, 2020). In the context of AI education, this orientation is particularly important because AI-related knowledge evolves rapidly and frequently renders prior learning outdated, creating a psychological cycle in which knowledge gaps intensify learners’ concerns about future competence and relevance (Wu and Li, 2025). Consequently, learners motivated by external pressures may be especially attentive to instructional environments that provide ongoing, up-to-date guidance rather than static, one-time content delivery.

Teacher support refers to the attitudes and behaviors teachers display toward students’ academic lives, encompassing both emotional support, such as care, encouragement, and respect, and academic support, such as commitment to learning and readiness to provide tangible assistance (Patrick et al., 2007; Shao et al., 2025). In digital and live-streamed contexts, these supportive functions also include course design and organization, facilitation of discussion, direct instruction, feedback, and technical guidance (Hu and Xiao, 2025). These elements overlap substantially with what the Community of Inquiry framework conceptualizes as teaching presence, which has been widely identified as a key factor in reducing transactional distance and sustaining participation in online learning environments (Garrison, 2016; Hu and Xiao, 2025).

In live-streamed AI courses, continuous teacher support is especially meaningful. Unlike pre-recorded or fully asynchronous formats, live-streamed instruction allows teachers to provide real-time explanations, iteratively update content in response to technological developments, address learner questions as they arise, and maintain an ongoing instructional presence across multiple sessions. Threat-related AI anxiety may also be positively associated with perceived continuous teacher support. Learners who experience stronger anxiety about AI-related uncertainty, obsolescence, and job displacement may become more attentive to instructional resources that reduce uncertainty and provide guidance (Wang et al., 2024; Wang and Wang, 2022; Wu and Li, 2025). In online learning environments, teacher support and teaching presence help learners cope with uncertainty and bridge transactional distance by offering clarification, responsiveness, and ongoing instructional direction (Garrison, 2016; Hu and Xiao, 2025). Accordingly, learners with higher threat-related AI anxiety may be more likely to notice, value, and rely on continuous teacher support in live-streamed AI courses as a coping resource and a psychologically safe pathway for managing technological threat (Shao et al., 2025; Wu and Li, 2025; Li et al., 2025). Therefore, the present study proposes:

*H2a*: Threat-related AI anxiety is positively associated with perceived continuous teacher support in live-streamed AI courses.

For learners with stronger extrinsic motivation for AI learning, these features are likely to be especially salient. Recent self-determination theory-based research suggests that learners driven by external and introjected regulations, such as avoiding the guilt of failure or the pressure of lagging behind, actively rely on supportive environments and facilitating conditions to achieve their goals (Li et al., 2025). Thus, extrinsically motivated learners are more likely to seek out, value, and utilize the continuous support offered by instructors in live-streamed AI courses, because such support provides a psychologically safe and more manageable pathway for responding to technological pressure and uncertainty (Wu and Li, 2025). Accordingly, the present study further proposes:

*H2b*: Extrinsic motivation for AI learning is positively associated with perceived continuous teacher support in live-streamed AI courses.

### 2.4 Engagement

Engagement in learning refers to the extent to which students actively participate in learning-related activities and is commonly understood as comprising behavioral, emotional, and cognitive dimensions (Fredricks et al., 2004). Behavioral engagement refers to active involvement in learning tasks, including effort, attention, and persistence. Emotional engagement reflects learners’ affective responses to the learning process. Cognitive engagement involves the use of metacognitive and self-regulatory strategies to deepen understanding and sustain learning (Fredricks et al., 2004; Hu and Xiao, 2025). This three-dimensional conceptualization has been widely adopted in online learning research and provides a useful framework for assessing the depth of learners’ participation (Reeve and Tseng, 2011; Sun and Rueda, 2012).

A substantial body of research has shown that teacher support is positively associated with learning engagement. From the perspective of self-determination theory (SDT), teacher support helps fulfill students’ basic psychological needs for autonomy, competence, and relatedness, thereby strengthening their willingness to invest in academic activities (Shao et al., 2025). Empirical evidence supports this reasoning. For example, Shao et al. (2025) found that teacher support was directly and positively associated with students’ learning engagement, with part of this relationship operating through academic self-efficacy and psychological resilience. In online learning environments, Hu and Xiao (2025) further concluded from a systematic review of 55 empirical studies that teaching presence—encompassing course design, discussion facilitation, direct instruction, and feedback—was the most frequently identified predictor of online learning engagement. Their review also showed that teacher behaviors such as providing multimodal feedback, hosting real-time interactive sessions, and offering clear guidance on technical issues can enhance learners’ involvement by reducing the transactional distance inherent in digital education (Hu and Xiao, 2025).

In live-streamed AI courses, perceived continuous teacher support may be especially important for engagement. AI-related content changes rapidly, and learners must often deal with complex concepts, steep learning curves, and uncertainty about what knowledge remains current and useful. Under these conditions, knowledge gaps can easily trigger anxiety and disengagement (Wu and Li, 2025). When learners perceive that instructors provide sustained guidance through repeated clarification, timely updates, and ongoing responsiveness, they are more likely to experience the learning process as manageable, trustworthy, and psychologically safe. As a result, they may be more willing to invest attention, effort, and active participation, thereby showing stronger behavioral, emotional, and cognitive engagement. This reasoning is consistent with Chiu (2022), who found that teacher support in AI-related learning contexts was significantly correlated with engagement, and with the broader conclusion of Hu and Xiao (2025) that perceived instructor support is a robust proximal predictor of online learning engagement across educational settings. Accordingly, the present study proposes:

*H3*: Perceived continuous teacher support is positively associated with engagement.

Self-determination theory has traditionally emphasized the stronger role of intrinsic motivation in fostering deep and sustained engagement (Deci and Ryan, 2000). At the same time, SDT also recognizes that extrinsic motivation, particularly when it is internalized to a greater degree, can support meaningful participation in learning activities (Ryan and Deci, 2020). In AI learning contexts, extrinsic motivation may be especially influential because the external demands that drive learners to study AI—such as occupational relevance, competitive pressure, and social expectations—are persistent and often perceived as legitimate (Wang et al., 2024). As a result, learners who are instrumentally motivated to acquire AI-related knowledge and skills may still invest substantial time, effort, and cognitive resources in learning activities, especially when those activities are closely aligned with urgent practical needs. Learners driven by external and introjected regulation actively rely on facilitating conditions to achieve their instrumental goals (Li et al., 2025). In live-streamed AI courses, where real-time instruction aligns closely with learners’ pressing needs, this positive association between extrinsic motivation and engagement may be especially pronounced (Hu and Xiao, 2025). Empirical evidence supports this reasoning. Wang et al. (2024) found that extrinsic motivation exerted a significant independent effect on AI learning intention even when intrinsic motivation was taken into account. Li et al. (2025) similarly showed that introjected and identified regulation were positively associated with behavioral engagement. Together, these findings suggest that externally regulated motivation can support meaningful involvement in AI learning. Accordingly, the present study proposes:

*H4*: Extrinsic motivation for AI learning is positively associated with engagement.

### 2.5 Mediating effects

Although extrinsic motivation may stimulate learners to participate in AI learning, it may not always be sufficient to sustain engagement on its own. This limitation is especially relevant in challenging and rapidly evolving learning domains such as AI, where learners often face steep learning curves, persistent uncertainty, and frequent knowledge obsolescence. Under such conditions, external pressure to learn AI may not be associated with active course participation unless learners also perceive that the instructional environment provides reliable and ongoing support. Without such support, the complexity of AI-related content and the resulting knowledge gaps can quickly turn external learning pressure into frustration, hesitation, or withdrawal (Wu and Li, 2025). Perceived continuous teacher support in live-streamed AI courses may therefore serve as a critical contextual bridge through which externally driven motivation is linked to active engagement. Extrinsically motivated learners are especially attentive to features of the learning environment that facilitate the attainment of instrumental goals (Ryan and Deci, 2020). In live-streamed AI courses, continuous teacher support represents precisely such a facilitating condition, providing ongoing guidance, feedback, and updated instruction that help learners manage the external demands motivating their participation. When extrinsically motivated learners perceive that this support is available, their externally driven motives become more actionable. By providing a psychologically safer and more manageable learning context, perceived continuous teacher support may help link motivational pressure to sustained behavioral, emotional, and cognitive engagement (Shao et al., 2025; Wu and Li, 2025). Accordingly, the present study proposes:

*H5*: Perceived continuous teacher support mediates the relationship between extrinsic motivation for AI learning and engagement.

More broadly, the preceding arguments suggest that the association between threat-related AI anxiety and engagement in live-streamed AI courses unfolds through a sequential process. First, threat-related AI anxiety heightens learners’ sense of urgency in responding to AI-related learning demands, thereby strengthening extrinsic motivation for AI learning (Teke, 2025; Wang et al., 2024). Second, because learners with stronger extrinsic motivation are more oriented toward supportive and performance-relevant features of the learning environment, they may become more sensitive to and more likely to value the continuous teacher support available in live-streamed AI courses (Li et al., 2025). Third, this perceived support helps reduce the transactional distance inherent in digital learning and promotes greater engagement by making the learning process more manageable, trustworthy, and psychologically safe (Hu and Xiao, 2025; Shao et al., 2025; Wu and Li, 2025). This serial pathway is particularly meaningful in applied AI education because it clarifies how a negative emotional condition may be linked to adaptive educational participation through motivational and contextual mechanisms. Rather than leading directly to disengagement, threat-related AI anxiety may activate externally driven motives that orient learners toward supportive live-streamed learning environments. By integrating the theoretical perspectives outlined in H1 through H5, the present study proposes a process account in which threat-related AI anxiety is associated with engagement through the sequential roles of extrinsic motivation and perceived continuous teacher support. Accordingly, the present study proposes:

*H6*: Threat-related AI anxiety has a positive serial indirect effect on engagement through extrinsic motivation for AI learning and perceived continuous teacher support.

### 2.6 Conditional effects of self-efficacy

Self-efficacy refers to an individual’s judgment of their capability to plan and execute courses of action required to attain designated goals (Bandura, 1977). It reflects not actual skill itself, but beliefs about what one can accomplish with the skills one possesses (Bandura, 1986). In educational contexts, self-efficacy has been consistently identified as an important predictor of learning engagement, persistence, and performance (Bandura, 2002; Brown, 2001; Compeau and Higgins, 1995; Shao et al., 2025). Learners with higher self-efficacy generally set more ambitious goals, invest greater effort, and persist more strongly when facing difficulty (Bandura, 2002; Shao et al., 2025).

In AI-related learning, self-efficacy is especially important because learners frequently encounter unfamiliar tools, rapidly changing content, and uncertainty regarding their own competence. Even when continuous teacher support is available, learners may differ substantially in the extent to which they can leverage that support for active engagement. Social cognitive theory suggests that self-efficacy beliefs shape how individuals interpret environmental resources and learning opportunities (Bandura, 1986). In remote and digital learning environments, learners with lower confidence often experience online interaction as more intimidating and are less likely to seek out opportunities to engage with instructors or peers (Lin et al., 2017; Teng et al., 2023). As a result, they may remain hesitant or passive even when instructional support is available. By contrast, learners with higher AI learning self-efficacy are more likely to perceive teacher support as actionable and useful because they feel capable of leveraging that support to overcome learning challenges.

This logic suggests that AI learning self-efficacy may moderate the relationship between perceived continuous teacher support and engagement in live-streamed AI courses. Although teacher support is generally expected to facilitate engagement, its marginal contribution may be weaker for learners with higher self-efficacy because these learners already possess stronger internal resources for sustaining participation. Empirical evidence is broadly consistent with this interpretation. Shao et al. (2025) found that academic self-efficacy helped explain how teacher support was linked to learning engagement, indicating that confidence in one’s capabilities is closely intertwined with the effectiveness of external support. In the present model, therefore, AI learning self-efficacy is expected to function as a compensatory boundary condition on the final stage of the pathway from perceived continuous teacher support to engagement. Accordingly, the present study proposes:

*H7*: AI learning self-efficacy weakens the positive relationship between perceived continuous teacher support and engagement.

**Figure 1:**
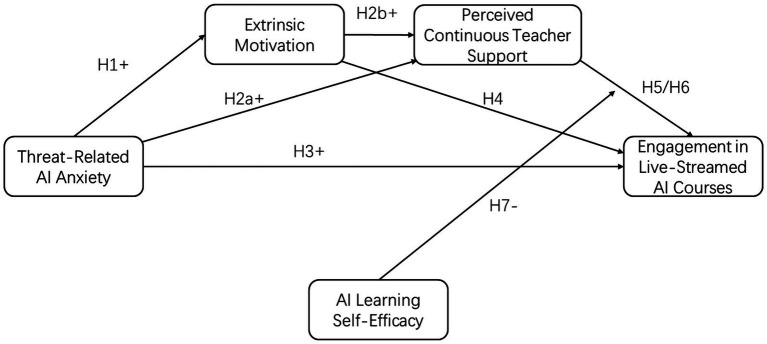
Theoretical model.

Figure 1 shows the theoretical model for this study.

## 3 Methods

### 3.1 Participants and procedure

The present study employed a cross-sectional, questionnaire-based survey design. The research context was commercial social media platforms in China, which have become a major channel for synchronous online AI education and skill development. These platforms host a large number of educational providers offering short-video-based promotional content and live-streamed AI courses, making them a highly relevant setting for examining learners’ engagement in live-streamed AI courses.

Ethical approval was obtained from the School of Journalism and Communication, Chongqing University, prior to data collection (CQUSJC2026007). All participants were provided with an informed consent form detailing the purpose of the study, ensuring the anonymity and confidentiality of their responses, and emphasizing their right to withdraw at any time without consequence. Participants were also informed that the data collected would be used solely for academic research purposes.

Participants were recruited by Chongqing Rounen Film and Television Media Co., Ltd. (Chongqing, China), a professional media market research company. To ensure sample appropriateness, only individuals who met the following eligibility criteria were included: (a) aged 18 years or older, (b) possessing basic awareness or usage experience of generative AI tools or applications, and (c) having actively participated in at least one live-streamed or other synchronous online AI-related course within the previous 6 months.

In line with recent methodological practices in AI education research (Wang et al., 2024), two screening questions were presented at the beginning of the survey to verify participant eligibility. The first assessed whether participants had basic awareness or usage experience of generative AI tools or applications, thereby ensuring that they could meaningfully evaluate AI-related concerns and learning demands. The second verified whether participants had recent experience participating in live-streamed or other synchronous online AI-related courses, which was necessary for accurately assessing perceived continuous teacher support and engagement in live-streamed AI courses.

After excluding incomplete questionnaires and responses that failed embedded attention-check items, the final valid sample consisted of 487 participants. Among them, 45.17% (*n* = 220) were male and 54.83% (*n* = 267) were female. In terms of age, 15.61% were aged 18–20, 38.81% were aged 21–25, 25.67% were aged 26–30, 13.35% were aged 31–40, and 6.57% were aged 41 or above. With respect to educational background, 6.16% had completed high school, 14.99% held a junior college diploma, 64.27% held a bachelor’s degree, and 14.58% held a master’s degree or above. To account for possible differences in technology-related learning experiences, participants’ field of study was also recorded: 47.02% came from STEM (science, technology, engineering, and mathematics) fields, whereas 52.98% came from non-STEM fields. Overall, the sample reflected diverse socioeconomic backgrounds and varying levels of prior AI familiarity. In addition, participants reported a mean of 12.08 months of prior AI experience (SD = 6.85), indicating substantial variation in previous exposure to AI tools and learning activities.

Several demographic and background variables were collected as potential covariates to reduce alternative explanations. Gender was considered because previous studies have shown that female learners may report higher levels of AI anxiety when facing complex AI technologies (Teke, 2025; Wu and Li, 2025). Field of study (STEM vs. non-STEM) was considered because non-STEM learners often report higher AI anxiety and lower levels of technological acceptance than their STEM counterparts (Li et al., 2025; Wu and Li, 2025). Prior AI experience and AI literacy were considered because previous exposure to AI may affect learners’ self-efficacy and cognitive anxiety (Hu and Xiao, 2025; Li et al., 2025; Teke, 2025). Socioeconomic status and family income were considered because economic conditions influence learners’ access to digital resources and their capacity to sustain online learning engagement (Li et al., 2025; Shao et al., 2025). Finally, age and educational background were considered because developmental stage and employment pressure may shape the relationship between threat-related AI anxiety, extrinsic motivation for AI learning, and engagement in live-streamed AI courses (Teke, 2025; Wu and Li, 2025).

Because all participants were Mandarin Chinese speakers, the survey was administered in Mandarin. To ensure measurement accuracy and equivalence, all scales originally developed in English were translated into Chinese using a rigorous forward-and-back-translation procedure. Two bilingual researchers independently conducted the translation and back-translation to ensure conceptual, linguistic, and cultural equivalence.

### 3.2 Measures

All constructs were measured using the mean scores of the corresponding items. All items were rated on a 7-point Likert scale ranging from 1 (strongly disagree) to 7 (strongly agree). The instruments were adapted from established scales and contextualized for live-streamed AI learning environments. The full set of adapted items is presented in the Supplementary material.

Threat-related AI anxiety was measured with seven items adapted from the Artificial Intelligence Anxiety Scale (AIAS) developed by Wang and Wang (2022), focusing on the job replacement anxiety and sociotechnical blindness dimensions. This operationalization was chosen because the present study centers on the threat-related component of AI anxiety that is most relevant to externally driven AI learning. A sample item is “I am afraid that AI will replace someone’s job.” The scale showed strong reliability and validity (Cronbach’s *α* = 0.957, M = 4.363, SD = 1.182, KMO = 0.954).

Extrinsic motivation for AI learning was measured with seven items adapted from Wang et al. (2024) and the AI Motivation Scale (AIMS) developed by Li et al. (2025). The items captured externally driven reasons for learning AI, including employability, competitiveness, salary enhancement, and goal utility. A sample item is “I think learning AI-related skills can increase my advantage in finding a job.” The scale demonstrated satisfactory psychometric properties (Cronbach’s *α* = 0.922, M = 4.885, SD = 0.885, KMO = 0.942).

Perceived continuous teacher support was measured with eight items adapted from the Teacher Support Scale originally developed by Johnson et al. (1983) and refined by Patrick et al. (2007). The original scale assesses general teacher support in classroom settings through items reflecting academic support (e.g., “My teacher helps me”) and emotional support (e.g., “My teacher treats me fairly”). To capture the continuous and real-time nature of support in live-streamed AI courses, the items were adapted in two ways. First, all references to classroom-based interaction were replaced with live-streamed instructional settings (e.g., “in this class” became “in the live-streamed AI courses”). Second, the wording was revised to emphasize the ongoing, iterative, and responsive nature of support characteristic of live-streamed instruction (e.g., “My teacher helps me” became “The instructor provides continuous help and explanations when I encounter difficulties with complex AI topics”; “My teacher treats me fairly” became “The instructor treats me fairly and creates a psychologically safe environment in the live streams”). The resulting items thus reflect not only general supportive attitudes but also the sustained availability, real-time responsiveness, and content-updating functions that distinguish teacher support in live-streamed AI courses from conventional classroom support. The scale showed good reliability and validity (Cronbach’s α = 0.933, M = 4.596, SD = 0.893, KMO = 0.954).

Engagement in live-streamed AI courses was measured using 12 items adapted from the engagement scale validated by Li et al. (2025), integrating behavioral, emotional, and cognitive engagement. The wording was contextualized for live-streamed AI learning. A sample item for behavioral engagement is “I actively participate in the interactive activities and discussions during the live-streamed AI courses”; for emotional engagement, “I enjoy learning new things about AI in the live-streamed courses”; and for cognitive engagement, “I actively think about how to apply the AI frameworks learned in the live streams to real-world problems.” The scale demonstrated excellent psychometric quality (Cronbach’s α = 0.969, M = 5.988, SD = 0.914, KMO = 0.980).

AI learning self-efficacy was measured with the five-item scale adopted from Wang et al. (2024), based on Bandura’s theory of self-efficacy. The scale assessed learners’ confidence in managing and mastering AI-related learning tasks. A sample item is “Learning AI-related skills is easy for me.” The measure showed strong reliability and validity (Cronbach’s α = 0.946, M = 4.457, SD = 1.260, KMO = 0.914).

### 3.3 Analytical strategy

Data analysis was conducted using IBM SPSS Statistics 29 and the PROCESS macro for SPSS (Hayes, 2022). Prior to hypothesis testing, descriptive statistics, Cronbach’s α coefficients, and Pearson correlations were computed for all study variables. To reduce multicollinearity in the moderation analyses, all continuous predictor variables were mean-centered before the interaction terms were created (Aiken and West, 1991).

PROCESS Model 87 was used because it provides a conditional process framework in which moderation is specified on the downstream paths from the mediators to the outcome, as well as on the resulting serial indirect effect. Across all regression equations, gender, age, educational background, and prior AI experience were included as covariates. Finally, a sensitivity analysis was conducted by dividing the sample into STEM and non-STEM subgroups. The same analytical procedures were then applied separately to each subgroup to assess the robustness of the proposed model across disciplinary backgrounds.

## 4 Results

### 4.1 Common method Bias assessment

Because all data were collected cross-sectionally through self-report questionnaires, common method bias was assessed before the main analyses (Podsakoff et al., 2003). Procedurally, the survey protected participant anonymity, reduced evaluation apprehension, and informed participants that there were no right or wrong answers, which helped reduce social desirability bias.

Statistically, Harman’s single-factor test was conducted using all 39 measurement items. The results yielded five factors with eigenvalues greater than 1.0, and the first unrotated factor accounted for 31.4% of the total variance, which was below the 50% threshold commonly used to indicate serious common method bias (Podsakoff et al., 2003). In addition, the correlations among the main constructs were moderate, with the highest correlation being between perceived continuous teacher support and engagement (*r* = 0.502, *p* < 0.01), which was well below the level typically associated with severe method bias. Variance inflation factor values also ranged from 1.02 to 1.53, below the conservative threshold of 3.3 suggested as a possible indicator of common method bias. Therefore, these results suggest that common method bias was unlikely to be a serious concern in the present study.

### 4.2 Preliminary analyses

Before testing the proposed hypotheses, we examined the reliability, validity, and preliminary associations among the study variables. As Table 1 showing, first, the measurement model showed good fit to the data, indicating that the five constructs were empirically distinguishable. All standardized factor loadings were significant and exceeded 0.70, ranging from 0.774 to 0.916.

**Table 1:** Preliminary analyses.

Variable	M	SD	α	1	2	3	4	5
1. Threat-related AI anxiety	4.363	1.182	0.957	—				
2. Extrinsic motivation for AI learning	4.885	0.885	0.922	0.289**	—			
3. Perceived continuous teacher support	4.596	0.893	0.933	0.075	0.457**	—		
4. AI learning self-efficacy	4.457	1.26	0.946	−0.290**	0.101*	0.048	—	
5. Engagement in live-streamed AI courses	5.988	0.914	0.969	0.197**	0.408**	0.502**	0.217**	—

*p < 0.05, **p < 0.01.

Second, all measures demonstrated satisfactory reliability and convergent validity. Cronbach’s alpha coefficients ranged from 0.922 to 0.969, composite reliability values ranged from 0.922 to 0.969, and average variance extracted values ranged from 0.628 to 0.780. These values exceeded commonly recommended thresholds, supporting internal consistency and convergent validity.

Third, discriminant validity was also supported. The square roots of the average variance extracted values were greater than the corresponding inter-construct correlations, and all heterotrait-monotrait ratios were below 0.85.

Fourth, descriptive statistics and bivariate correlations are presented in Table 1. Threat-related AI anxiety was positively associated with extrinsic motivation for AI learning (*r* = 0.289, *p* < 0.01) and engagement in live-streamed AI courses (*r* = 0.197, *p* < 0.01), and negatively associated with AI learning self-efficacy (*r* = −0.290, *p* < 0.01). Extrinsic motivation for AI learning was positively associated with perceived continuous teacher support (*r* = 0.457, *p* < 0.01), AI learning self-efficacy (*r* = 0.101, *p* < 0.05), and engagement (*r* = 0.408, *p* < 0.01). Perceived continuous teacher support was also positively associated with engagement (*r* = 0.502, *p* < 0.01). AI learning self-efficacy was positively associated with engagement as well (*r* = 0.217, *p* < 0.01). These preliminary results supported the adequacy of the measurement model and were generally consistent with the hypothesized relationships, thereby justifying the subsequent moderated serial mediation analysis.

### 4.3 Hypothesis testing

Table 2 presents the regression results from the moderated serial mediation analysis (PROCESS Model 87). Table 3 reports the indirect and conditional indirect effects, and Table 4 presents the conditional effects of perceived continuous teacher support on engagement at different levels of AI learning self-efficacy. All models included gender, age, educational background, and prior AI experience as covariates, and all continuous variables were mean-centered prior to analysis.

**Table 2:** Regression results for the moderated serial mediation model (PROCESS model 87).

Pathway	*B*	*SE*	*t*	*p*	95% CI
Outcome: Extrinsic motivation for AI learning (*R*^2^ = 0.091)					
Threat-related AI anxiety → Extrinsic motivation	0.22	0.03	6.59	<0.001	[0.15, 0.28]
Outcome: Perceived continuous teacher support (*R*^2^ = 0.217)					
Threat-related AI anxiety → Teacher support	−0.05	0.03	−1.58	0.115	[−0.11, 0.01]
Extrinsic motivation → Teacher support	0.48	0.04	11.32	<0.001	[0.40, 0.57]
Outcome: Engagement (*R*^2^ = 0.370)					
Threat-related AI anxiety → Engagement	0.15	0.03	5.40	<0.001	[0.10, 0.21]
Extrinsic motivation → Engagement	0.13	0.04	3.05	0.002	[0.05, 0.22]
Teacher support → Engagement	0.41	0.04	9.45	<0.001	[0.33, 0.50]
AI learning self-efficacy → Engagement	0.18	0.03	5.72	<0.001	[0.12, 0.24]
Teacher support * Self-efficacy → Engagement	−0.10	0.03	−3.57	<0.001	[−0.15, −0.04]

Unstandardized coefficients with heteroskedasticity-consistent (HC3) standard errors. CI, confidence interval.

**Table 3:** Indirect and conditional indirect effects of threat-related AI anxiety on engagement.

Indirect pathway	Self-efficacy	Effect	*Boot SE*	Boot LLCI	Boot ULCI	Sig.
Threat-related AI anxiety → Extrinsic motivation → Engagement						
	(unconditional)	0.03	0.01	0.01	0.05	Yes
Threat-related AI anxiety → Teacher support → Engagement						
	Low (−1 SD)	−0.03	0.02	−0.06	0.006	No
	Mean	−0.02	0.01	−0.05	0.005	No
	High (+1 SD)	−0.01	0.01	−0.04	0.003	No
*Index of moderated mediation*		0.005	0.004	−0.001	0.013	No
Threat-related AI anxiety → Extrinsic motivation → Teacher support → Engagement						
	Low (−1 SD)	0.06	0.01	0.04	0.08	Yes
	Mean	0.04	0.01	0.03	0.06	Yes
	High (+1 SD)	0.03	0.01	0.02	0.05	Yes
*Index of moderated mediation*		−0.010	0.003	−0.017	−0.004	Yes

Effects estimated via 5,000 bootstrap samples. Self-efficacy levels correspond to ±1 standard deviation from the mean (Low = −1.26, High = +1.26). “Sig.” indicates whether the 95% bootstrap confidence interval excludes zero.

**Table 4:** Conditional effects of perceived continuous teacher support on engagement at different levels of AI learning self-efficacy.

Pathway	Low (−1 SD)	Mean	High (+1 SD)
Teacher support → Engagement	0.53 [0.43, 0.64]	0.41 [0.33, 0.50]	0.29 [0.18, 0.40]

Conditional effects (unstandardized) with 95% confidence intervals in brackets. Low and high self-efficacy correspond to ±1 standard deviation from the mean.

H1 proposed that threat-related AI anxiety would be positively associated with extrinsic motivation for AI learning. As shown in Table 2, threat-related AI anxiety was positively associated with extrinsic motivation (*B* = 0.22, *p* < 0.001, 95% CI [0.15, 0.28]). Therefore, H1 was supported.

H2a proposed that threat-related AI anxiety would be positively associated with perceived continuous teacher support in live-streamed AI courses. As shown in Table 2, threat-related AI anxiety was not significantly associated with perceived continuous teacher support (*B* = −0.05, *p* = 0.115, 95% CI [−0.11, 0.01]). Therefore, H2a was not supported.

H2b proposed that extrinsic motivation for AI learning would be positively associated with perceived continuous teacher support in live-streamed AI courses. As shown in Table 2, extrinsic motivation was positively associated with perceived continuous teacher support (*B* = 0.48, *p* < 0.001, 95% CI [0.40, 0.57]). Therefore, H2b was supported.

H3 proposed that perceived continuous teacher support would be positively associated with engagement in live-streamed AI courses. As shown in Table 2, perceived continuous teacher support was positively associated with engagement (*B* = 0.41, *p* < 0.001, 95% CI [0.33, 0.50]). Therefore, H3 was supported.

H4 proposed that extrinsic motivation for AI learning would be positively associated with engagement in live-streamed AI courses. As shown in Table 2, extrinsic motivation was positively associated with engagement (*B* = 0.13, *p* = 0.002, 95% CI [0.05, 0.22]). Therefore, H4 was supported.

H5 proposed that perceived continuous teacher support would mediate the relationship between extrinsic motivation for AI learning and engagement. As shown in Table 3, the indirect effect of threat-related AI anxiety on engagement through perceived continuous teacher support alone was not significant at any level of AI learning self-efficacy (e.g., at the mean: Effect = −0.02, 95% Boot CI [−0.05, 0.005]). However, the pathway from extrinsic motivation to engagement through perceived continuous teacher support remained meaningful as part of the broader serial mediation process. Thus, the mediating role of perceived continuous teacher support was supported within the serial mechanism, although the simple indirect path from threat-related AI anxiety through perceived continuous teacher support alone was not supported.

H6 proposed that threat-related AI anxiety would have a positive serial indirect association with engagement through extrinsic motivation for AI learning and perceived continuous teacher support. As shown in Table 3, the serial indirect effect was significant at low AI learning self-efficacy (Effect = 0.06, 95% Boot CI [0.04, 0.08]), mean AI learning self-efficacy (Effect = 0.04, 95% Boot CI [0.03, 0.06]), and high AI learning self-efficacy (Effect = 0.03, 95% Boot CI [0.02, 0.05]). The effect was significant at all levels, indicating a robust serial indirect pathway. Therefore, H6 was supported.

**Figure 2:**
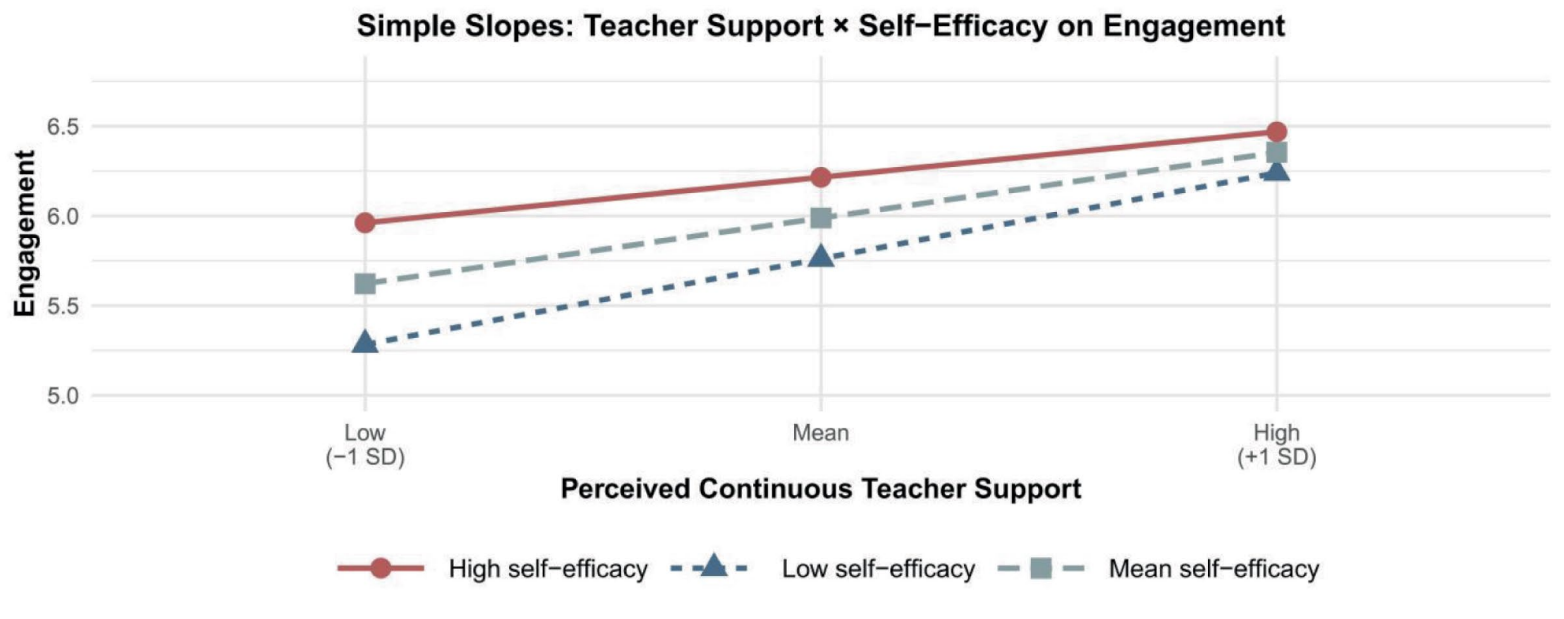
Simple slope.

H7 proposed that AI learning self-efficacy would weaken the positive association between perceived continuous teacher support and engagement. As shown in Table 2, the interaction between perceived continuous teacher support and AI learning self-efficacy was significant (*B* = −0.10, *p* < 0.001, 95% CI [−0.15, −0.04]). The conditional effects (Table 4; Figure 2) showed that the association between perceived continuous teacher support and engagement was strongest at low AI learning self-efficacy (Effect = 0.53, *p* < 0.001, 95% CI [0.43, 0.64]), weaker at mean AI learning self-efficacy (Effect = 0.41, *p* < 0.001, 95% CI [0.33, 0.50]), and weakest at high AI learning self-efficacy (Effect = 0.29, *p* < 0.001, 95% CI [0.18, 0.40]). Therefore, H7 was supported.

**Figure 3:**
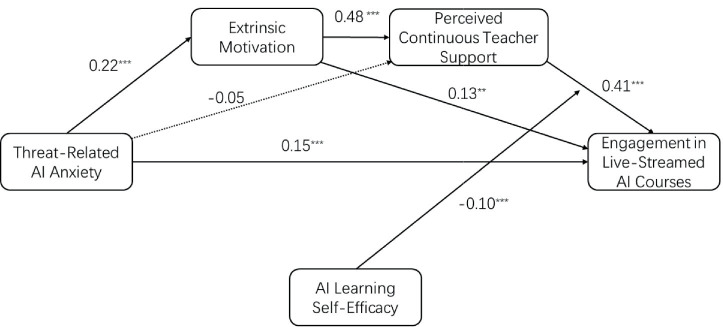
Pathway. Solid lines indicate significance; dashed lines indicate no significance.

Figure 3 shows the path coefficients in this study.

### 4.4 Robustness

The model explained 9% of the variance in extrinsic motivation for AI learning, 22% of the variance in perceived continuous teacher support, and 37% of the variance in engagement. Robust Breusch–Pagan tests indicated no evidence of heteroskedasticity for extrinsic motivation (*χ*^2^ = 2.55, *p* = 0.769) or perceived continuous teacher support (*χ*^2^ = 8.91, *p* = 0.179). For the engagement equation, the robust test was also not significant (*χ*^2^ = 11.57, *p* = 0.239). To ensure the maximum robustness of the estimates, all models were estimated utilizing heteroskedasticity-consistent standard errors.

Variance inflation factors across all equations ranged from 1.01 to 1.45, well below the conventional threshold of 3.0, indicating that multicollinearity was not a concern.

### 4.5 Sensitivity analysis

To examine whether the proposed model was robust across disciplinary backgrounds, the analyses were repeated separately for participants from non-STEM fields (*n* = 258) and STEM fields (*n* = 229).

Among participants from non-STEM fields, the core serial mediation pattern remained evident. Threat-related AI anxiety was positively associated with extrinsic motivation for AI learning (*B* = 0.45, *p* < 0.001), and extrinsic motivation was positively associated with perceived continuous teacher support (*B* = 0.54, *p* < 0.001). In the engagement equation, both extrinsic motivation (*B* = 0.18, *p* < 0.001) and perceived continuous teacher support (*B* = 0.54, *p* < 0.001) were positively associated with engagement. The serial indirect effect through extrinsic motivation and perceived continuous teacher support was significant at low, mean, and high levels of AI learning self-efficacy (Effects = 0.14, 0.13, and 0.12, respectively; all bootstrap confidence intervals excluded zero). However, threat-related AI anxiety was not significantly associated with perceived continuous teacher support (*B* = −0.10, *p* = 0.067), and its direct association with engagement was not significant (*B* = −0.01, *p* = 0.799). The interaction between perceived continuous teacher support and AI learning self-efficacy was not significant in this subgroup (*B* = −0.04, *p* = 0.204), and the index of moderated mediation for the serial indirect effect included zero (Index = −0.010, 95% Boot CI [−0.028, 0.005]).

Among participants from STEM fields, the same core serial mediation mechanism was observed. Threat-related AI anxiety was positively associated with extrinsic motivation for AI learning (*B* = 0.28, *p* < 0.001), and extrinsic motivation was positively associated with perceived continuous teacher support (*B* = 0.34, *p* < 0.001). In the engagement equation, both extrinsic motivation (*B* = 0.35, *p* < 0.001) and perceived continuous teacher support (*B* = 0.35, *p* < 0.001) were positively associated with engagement. The serial indirect effect was significant at all levels of self-efficacy (Effects = 0.04, 0.03, and 0.03, respectively; all bootstrap confidence intervals excluded zero). Threat-related AI anxiety was not significantly associated with perceived continuous teacher support (*B* = 0.03, *p* = 0.548), and its direct association with engagement was not significant (*B* = −0.06, *p* = 0.194). The interaction between perceived continuous teacher support and AI learning self-efficacy was marginally non-significant (*B* = −0.10, *p* = 0.094), and the index of moderated mediation for the serial indirect effect included zero (Index = −0.010, 95% Boot CI [−0.025, 0.001]).

These subgroup analyses indicate that the core serial mediation mechanism was stable across disciplinary backgrounds: in both non-STEM and STEM groups, threat-related AI anxiety was linked to higher extrinsic motivation, extrinsic motivation was linked to stronger perceived continuous teacher support, and the serial indirect association with engagement remained significant. However, the moderating effect of AI learning self-efficacy on the association between perceived continuous teacher support and engagement, which was significant in the full sample, did not reach significance in either subgroup. This may reflect reduced statistical power in the smaller subsamples, as the interaction coefficient in the STEM subgroup was of similar magnitude to the full sample but did not reach conventional significance levels. These findings suggest that the central serial mediation process generalizes across fields of study, whereas the conditional role of self-efficacy may require larger samples to detect reliably at the subgroup level.

## 5 Discussion

This study examined how threat-related AI anxiety is associated with learners’ engagement in live-streamed AI courses through extrinsic motivation for AI learning and perceived continuous teacher support, and whether the downstream link from perceived continuous teacher support to engagement depends on AI learning self-efficacy. The focus on live-streamed AI courses is important because practical AI education is often delivered in this format. AI-related tools, prompts, platforms, and workflows change rapidly, so learners frequently need instruction that can be updated in real time, explained step by step, and supported through immediate interaction (Hu and Xiao, 2025; Wu and Li, 2025). In this sense, live-streamed AI courses are well suited to applied AI education because they combine flexibility, timeliness, and continuing instructional support (Hu and Xiao, 2025).

Several main findings emerged. First, threat-related AI anxiety was positively associated with extrinsic motivation for AI learning, indicating that AI-related threat may activate externally driven learning motives (Teke, 2025; Wang et al., 2024). Second, extrinsic motivation was positively associated with perceived continuous teacher support, and perceived continuous teacher support was positively associated with engagement in live-streamed AI courses (Hu and Xiao, 2025; Li et al., 2025; Shao et al., 2025). Third, the analyses supported a significant serial indirect association of threat-related AI anxiety with engagement through extrinsic motivation and perceived continuous teacher support. Fourth, AI learning self-efficacy significantly weakened the association between perceived continuous teacher support and engagement, indicating that learners with lower self-efficacy depended more heavily on instructional support for their engagement. At the same time, threat-related AI anxiety was not significantly associated with perceived continuous teacher support (H2a), suggesting that anxiety is linked to engagement through motivational activation rather than through directly heightening perceptions of teacher support. These results suggest that emotional pressure is linked to engagement primarily through motivational activation followed by a supportive instructional process, and that the effectiveness of instructional support in sustaining engagement is associated with learners’ internal confidence.

The findings contribute to theory in several ways. First, the study advances research on AI anxiety by showing that threat-related AI anxiety may function as an activating emotional force in applied AI education. In line with the Control-Value Theory of Achievement Emotions, anxiety may be associated with approach-oriented behavior when learners perceive the task as important and consequential (Pekrun, 2006). This interpretation is also consistent with the distinction between activating and deactivating negative emotions in achievement settings. Consistent with Wang et al. (2024), the present findings show that threat-related dimensions of AI anxiety are positively associated with extrinsic motivation for AI learning. The results also resonate with Teke (2025), who found that learners with stronger AI risk-awareness displayed higher levels of motivated learning behavior. Building on this line of research, the present study extends the literature by showing that the activating role of AI anxiety does not end at motivational activation. As recent systematic reviews emphasize, breaking the cycle of technology-induced fear requires transforming existential threats to professional identity into proactive learner agency (Wu and Li, 2025). The present findings suggest that this process may unfold through a broader pathway that reaches actual engagement in live-streamed AI courses.

Second, the study contributes to theory by identifying perceived continuous teacher support as a central instructional mechanism through which extrinsic motivation is linked to engagement. In line with self-determination theory, learners with externally regulated motives orient themselves toward contextual conditions that help them attain valued goals (Deci and Ryan, 2000; Ryan and Deci, 2020). This logic is also consistent with recent work by Li et al. (2025), which suggests that learners under external and introjected pressure actively seek facilitating conditions to cope with AI-related demands and avoid the guilt of falling behind peers. In line with prior research on online engagement, the present findings support the view that teacher support and teaching presence are closely associated with sustained participation in digital learning environments by bridging the inherent transactional distance (Hu and Xiao, 2025; Shao et al., 2025). What the present study adds is a more specific process explanation situated in live-streamed AI courses. It shows that continuous teacher support is not merely an accompanying contextual feature. Rather, it functions as a psychologically meaningful mechanism through which motivational pressure is linked to sustained engagement.

At the same time, the non-significant path from threat-related AI anxiety to perceived continuous teacher support adds an important nuance. Theoretically, it seemed plausible that anxious learners would become more attentive to teacher guidance as a coping resource. However, the results suggest that anxiety by itself may not be sufficient to heighten perceived teacher support. Instead, anxiety appears to be linked to engagement more indirectly, first through its association with extrinsic motivation, which in turn is associated with learners’ orientation toward instructional support. In other words, learners may not perceive more support simply because they feel threatened; rather, they may become more likely to notice, value, and utilize teacher support when that threat is channeled into a concrete motivational intention to learn AI. This interpretation is theoretically consistent with self-determination theory, which emphasizes that externally driven motives shape how learners engage with environmental affordances (Ryan and Deci, 2020; Li et al., 2025), and with online learning research showing that teaching presence is associated with engagement when learners actively orient themselves toward the instructional environment (Garrison, 2016; Hu and Xiao, 2025). Thus, the unsupported direct path from anxiety to perceived support does not undermine the broader framework, but instead refines it by suggesting that motivation is the key mechanism through which emotional pressure becomes linked to supportive instructional perception.

Third, the study refines theory on AI learning self-efficacy by showing that it functions as a boundary condition on the final stage of the serial pathway—specifically, the link from perceived continuous teacher support to engagement. In line with social cognitive theory, self-efficacy shapes how learners interpret demands, mobilize effort, and use available resources (Bandura, 1977, 1986). Prior studies have suggested that self-efficacy is deeply involved in AI learning processes and digital learning adaptation (Teng et al., 2023; Wang et al., 2024), and systematic reviews further emphasize that self-efficacy is a core determinant of whether students actively engage with or avoid technologically demanding educational environments (Hu and Xiao, 2025; Wu and Li, 2025). In the present study, AI learning self-efficacy significantly weakened the positive association between perceived continuous teacher support and engagement (*B* = −0.10, *p* < 0.001). The conditional effects showed that the association between teacher support and engagement was nearly twice as strong at low self-efficacy (0.53) as at high self-efficacy (0.29). This means that learners with lower AI learning self-efficacy depended more heavily on continuous teacher support for their engagement, whereas learners with higher self-efficacy were able to maintain engagement even when teacher support was less salient.

This finding is theoretically meaningful. Learners with lower AI learning self-efficacy may depend more heavily on teacher support because external guidance may help reduce uncertainty, structure difficult content, and compensate for weak internal confidence. By contrast, learners with higher self-efficacy may be better able to regulate their own learning and therefore rely less on continuous teacher support to remain engaged. This interpretation aligns with prior work showing that self-efficacy is associated with how learners use available resources in digital learning environments (Teng et al., 2023; Shao et al., 2025). Notably, the serial indirect effect through extrinsic motivation and perceived continuous teacher support was also stronger at lower self-efficacy (0.06) than at higher self-efficacy (0.03), and the index of moderated mediation for this serial path was significant (Index = −0.010, 95% Boot CI [−0.017, −0.004]). This indicates that the full externally driven pathway—from anxiety-driven motivation, through teacher support, to engagement—was more consequential for learners with lower internal confidence.

It should also be acknowledged that the model explained only approximately 9% of the variance in extrinsic motivation for AI learning. While the association between threat-related AI anxiety and extrinsic motivation was statistically significant and theoretically meaningful, this limited explanatory power suggests that important additional variables were not included in the present model. Factors such as intrinsic motivation, perceived usefulness of AI, career identity salience, labor market perceptions, and institutional incentive structures may all contribute meaningfully to extrinsic motivation for AI learning (Li et al., 2025; Wang et al., 2024). Future research should consider expanding the variable set to provide a more comprehensive account of the motivational antecedents of AI learning.

The sensitivity analysis provides additional insight into these patterns. Across both non-STEM and STEM subgroups, the core serial mediation mechanism remained stable: threat-related AI anxiety was positively associated with extrinsic motivation for AI learning, extrinsic motivation was positively associated with perceived continuous teacher support, and the serial indirect effect on engagement was significant in both groups. This strengthens confidence in the central process model and suggests that the pathway from emotional pressure to motivation, support, and engagement is not restricted to one disciplinary background.

At the same time, the moderating effect of AI learning self-efficacy on the association between perceived continuous teacher support and engagement did not reach significance in either subgroup (non-STEM: B = −0.04, *p* = 0.204; STEM: B = −0.10, *p* = 0.094). This non-replication at the subgroup level may reflect reduced statistical power, as the STEM subgroup coefficient (−0.10) was of similar magnitude to the full-sample estimate (−0.10) but fell short of significance in the smaller sample (*n* = 229). The non-STEM subgroup showed a notably weaker coefficient (−0.04), suggesting that in this group, the conditional importance of self-efficacy for the teacher support–engagement link may be less pronounced. One possible interpretation is that non-STEM learners’ engagement was so strongly associated with teacher support regardless of self-efficacy (B = 0.54 unconditionally) that individual differences in self-efficacy had less room to moderate this relationship. By contrast, in the STEM subgroup, teacher support was less strongly associated with engagement overall (B = 0.35), potentially leaving more scope for self-efficacy to differentiate learners’ responses to instructional support. These subgroup differences should be interpreted cautiously given the sample sizes, but they suggest that the conditional role of self-efficacy may operate differently across disciplinary contexts.

The present findings offer several practical implications for live-streamed AI education. First, they suggest that live-streamed AI courses should be designed as psychologically responsive learning environments. In line with previous research showing the importance of teacher support and teaching presence in online learning (Hu and Xiao, 2025; Shao et al., 2025), the current results indicate that continuous teacher support is centrally associated with sustained engagement by bridging transactional distance. Course designers and instructors should therefore provide ongoing explanation, timely feedback, repeated clarification, and visible instructional presence throughout the learning process. By doing so, educators can create psychologically safe pathways where emotional and academic support converge, helping learners manage technological uncertainty and remain actively involved (Wu and Li, 2025).

Second, the findings suggest that AI anxiety should be managed as a motivational resource as well as a psychological challenge. In line with recent evidence (Teke, 2025; Wang et al., 2024), the results show that threat-related AI anxiety is associated with engagement through extrinsic motivation and teacher support. Rather than merely attempting to eliminate technology-induced fears, educators should recognize that concerns about job replacement, obsolescence, and AI-related uncertainty may be associated with adaptive educational behavior when they are channeled into clear learning goals, structured progression paths, and manageable course tasks. In practice, this means that instructors should acknowledge learners’ concerns while helping them channel those concerns into concrete reasons for participation and skill development.

Third, the results are especially relevant for learners with lower AI learning self-efficacy. Because teacher support proved particularly consequential where internal confidence was weaker—with the association between teacher support and engagement being nearly twice as strong at low self-efficacy as at high self-efficacy—live-streamed AI courses should include low-threshold entry points, scaffolded instruction, incremental mastery experiences, and supportive feedback (Wu and Li, 2025). These practices may help reduce hesitation in digital learning environments, support continued engagement, and gradually strengthen learners’ capacity for self-regulated participation (Teng et al., 2023).

This study has several limitations. First, the cross-sectional design limits strong causal inference. Although the proposed model was grounded in established theories, the temporal ordering among threat-related AI anxiety, extrinsic motivation for AI learning, perceived continuous teacher support, AI learning self-efficacy, and engagement cannot be fully confirmed. In line with prior AI learning research that has also relied heavily on cross-sectional evidence (Teke, 2025; Wang et al., 2024), the present study identifies theoretically meaningful associations, but future research should use longitudinal or experimental designs to test causal development and dynamic relationships over time more directly.

Second, all variables were measured through self-report, which is common in studies of anxiety, motivation, and self-efficacy, but may not fully capture actual learning behavior. In line with previous online learning research (Hu and Xiao, 2025; Li et al., 2025), the present study focused on learners’ subjective perceptions. Future studies could combine self-report data with multimodal behavioral indicators, such as attendance, interaction records, or learning management system data, as well as direct teacher observational assessments of student engagement, to provide a fuller and more objective account of engagement in live-streamed AI courses.

Third, the direct path from threat-related AI anxiety to perceived continuous teacher support was not supported. This non-significant finding suggests that the proposed conditional process model may be more selective than initially expected. Future research should examine whether additional contextual or individual factors, such as perceived relevance of AI, prior AI literacy, or specific instructional features of live-streamed courses, help explain when and for whom anxiety is more directly linked to perceptions of teacher support. It should also be acknowledged that the model explained only approximately 9% of the variance in extrinsic motivation for AI learning. While the association between threat-related AI anxiety and extrinsic motivation was statistically significant and theoretically meaningful, this limited explanatory power suggests that important additional predictors were not included in the present model. Factors such as intrinsic motivation, perceived usefulness of AI, career identity salience, labor market perceptions, and institutional incentive structures may all contribute meaningfully to extrinsic motivation for AI learning (Li et al., 2025; Wang et al., 2024). Future research should consider expanding the predictor set to provide a more comprehensive account of the motivational antecedents of AI learning.

Fourth, the moderating effect of AI learning self-efficacy on the association between perceived continuous teacher support and engagement was significant in the full sample but did not replicate in either subgroup. This may reflect reduced statistical power in the smaller subsamples, or it may indicate that the moderation effect is relatively modest and requires larger samples to detect reliably. Future research with larger disciplinary subgroups could clarify whether this boundary condition is robust across different learner populations.

Fifth, the distinction between STEM and non-STEM fields was useful for the sensitivity analysis, but it remained broad. Prior research suggests that learners from different disciplines vary in digital confidence, AI relevance, and prior exposure (Li et al., 2025; Wu and Li, 2025). Future research could adopt more fine-grained disciplinary categories and examine whether more specific background factors, such as prior AI experience, technological identity, or occupational expectations, explain more variance than broad disciplinary grouping alone.

Sixth, participants were recruited through a professional media research company and were required to have recent experience with live-streamed AI courses. This eligibility requirement likely introduced self-selection bias, as learners who actively seek out and participate in such courses may possess higher baseline motivation, greater openness to AI-related learning, or lower anxiety thresholds than the broader population of potential AI learners. As a result, the present findings may underestimate the strength of AI anxiety effects or overestimate engagement levels relative to more representative samples. Future research should consider broader recruitment strategies, including outreach to learners who have not yet participated in AI courses, to improve the generalizability of findings.

## 6 Conclusion

As artificial intelligence continues to reshape learning and work, live-streamed AI courses have become an important format for timely, interactive, and practice-oriented instruction. This study shows that threat-related AI anxiety is positively associated with engagement in these courses primarily through a serial pathway involving extrinsic motivation for AI learning and perceived continuous teacher support. AI learning self-efficacy weakened the positive association between perceived continuous teacher support and engagement, such that the association between teacher support and engagement was nearly twice as strong at low self-efficacy as at high self-efficacy. The serial indirect pathway through extrinsic motivation and perceived continuous teacher support was also more pronounced at lower levels of self-efficacy, as indicated by a significant index of moderated mediation. However, the direct path from threat-related AI anxiety to perceived continuous teacher support was not supported, and the moderating effect of self-efficacy did not replicate in either the STEM or non-STEM subgroup, possibly reflecting reduced statistical power in the smaller subsamples. Overall, the findings suggest that learners’ responses to AI-related educational pressure reflect the combined associations among emotional, motivational, instructional, and self-regulatory processes, highlighting the value of designing live-streamed AI courses with clear guidance, continuous support, and accessible entry points, particularly for learners with lower confidence in their AI learning capabilities.

## Data Availability

The raw data supporting the conclusions of this article will be made available by the authors, without undue reservation.
