# Supplementary material for: Threat-related AI anxiety and engagement in live-streamed AI courses: a moderated serial mediation model of extrinsic motivation and teacher support
Source: Front Psychol. 2026 Apr 29;17:1831785. doi: 10.3389/fpsyg.2026.1831785 (PMC13168054; doi:10.3389/fpsyg.2026.1831785)
Supplement: Supplementary file 1 [file Supplementary_file_1.docx]

Supplementary Material

# Threat-Related AI Anxiety (AIA)

| **Construct** | **Item** | **Measurement Item (English)** | **Mean** | **SD** |
| --- | --- | --- | --- | --- |
| Threat-Related AI Anxiety (AIA) | AIA1 | I am afraid that AI may replace humans. | 4.39 | 1.327 |
|  | AIA2 | I am afraid that widespread use of humanoid robots will take jobs away from people. | 4.353 | 1.347 |
|  | AIA3 | I am afraid that AI will replace someone’s job. | 4.306 | 1.339 |
|  | AIA4 | I am afraid that an AI technique/product may be misused. | 4.384 | 1.284 |
|  | AIA5 | I am afraid of various problems potentially associated with an AI technique/product. | 4.363 | 1.326 |
|  | AIA6 | I am afraid that an AI technique/product may get out of control and malfunction. | 4.378 | 1.299 |
|  | AIA7 | I am afraid that an AI technique/product may lead to robot autonomy. | 4.368 | 1.359 |
| *AIA Construct Average* | | *Overall Threat-Related AI Anxiety* | *4.363* | *1.182* |
| Extrinsic Motivation for AI Learning (EM) | EM1 | I think learning AI-related skills can increase my advantage in finding a job. | 4.881 | 1.076 |
|  | EM2 | I think learning AI-related skills can ensure that I keep my job in the future. | 4.867 | 1.081 |
|  | EM3 | I think learning AI-related skills can help me get a higher salary in the future. | 4.897 | 1.084 |
|  | EM4 | I think my opinions will receive more attention from the team after learning AI-related skills. | 4.916 | 1.063 |
|  | EM5 | I think learning AI-related skills improves people’s opinion of me. | 4.864 | 1.105 |
|  | EM6 | I will be recognized for my outstanding performance in learning AI-related skills. | 4.869 | 1.084 |
|  | EM7 | In general, I think learning AI-related skills is very useful for achieving my goals. | 4.899 | 1.014 |
| *EM Construct Average* | | *Overall Extrinsic Motivation for AI Learning* | *4.885* | *0.885* |
| Perceived Continuous Teacher Support (PTS) | PTS1 | In the live-streamed AI courses, the instructor continuously encourages me to do my best. | 4.602 | 1.076 |
|  | PTS2 | The instructor provides continuous help and explanations when I encounter difficulties with complex AI topics. | 4.577 | 1.076 |
|  | PTS3 | The instructor consistently checks to see if we understand the rapidly updating AI content. | 4.585 | 1.07 |
|  | PTS4 | The instructor is always ready to provide tangible assistance and real-time feedback during the live streams. | 4.573 | 1.082 |
|  | PTS5 | In the live-streamed AI courses, the instructor really understands how I feel about learning AI. | 4.604 | 1.08 |
|  | PTS6 | The instructor continuously shows care and respect for my questions as they emerge in real-time. | 4.591 | 1.079 |
|  | PTS7 | The instructor treats me fairly and creates a psychologically safe environment in the live streams. | 4.608 | 1.111 |
|  | PTS8 | I feel the instructor is consistently committed to supporting my overall learning experience. | 4.628 | 1.088 |
| *PTS Construct Average* | | *Overall Perceived Continuous Teacher Support* | *4.596* | *0.893* |
| AI Learning Self-Efficacy (SE) | SE1 | Learning AI-related skills is easy for me. | 4.444 | 1.425 |
|  | SE2 | I do not think I lack the ability to learn AI-related skills. | 4.476 | 1.424 |
|  | SE3 | I do not think I lack the foundation for AI-related skills learning. | 4.448 | 1.355 |
|  | SE4 | I am not afraid to learn AI-related skills. | 4.503 | 1.338 |
|  | SE5 | I have enough intelligence to learn AI-related skills. | 4.417 | 1.397 |
| *SE Construct Average* | | *Overall AI Learning Self-Efficacy* | *4.457* | *1.26* |
| Engagement in Live-Streamed AI Courses (ENG) | ENG1 | I can consistently maintain focus during the live-streamed AI courses. | 5.975 | 1.044 |
|  | ENG2 | I actively participate in the interactive activities and discussions during the live-streamed AI courses. | 6.045 | 1.049 |
|  | ENG3 | I put a lot of effort into understanding the AI concepts taught in the live streams. | 5.971 | 1.058 |
|  | ENG4 | I pay careful attention when the instructor explains complex AI tools or algorithms in real-time. | 5.979 | 1.09 |
|  | ENG5 | I enjoy learning new things about AI in the live-streamed courses. | 5.975 | 1.092 |
|  | ENG6 | I find the live-streamed AI courses to be interesting and engaging. | 5.945 | 1.061 |
|  | ENG7 | I feel excited when we explore new AI applications during the live streams. | 5.99 | 1.069 |
|  | ENG8 | I feel positive and enthusiastic about participating in these live-streamed AI sessions. | 5.998 | 0.997 |
|  | ENG9 | When learning in the live-streamed AI courses, I try to relate what I’m learning to what I already know. | 6.06 | 1.022 |
|  | ENG10 | I actively think about how to apply the AI frameworks learned in the live streams to real-world problems. | 5.969 | 1.049 |
|  | ENG11 | I consistently monitor my own understanding of the rapidly updating AI content during the course. | 5.971 | 1.063 |
|  | ENG12 | When I encounter difficult AI tasks in the live streams, I try to figure them out by using different learning strategies. | 5.982 | 1.08 |
| *ENG Construct Average* | | *Overall Engagement in Live-Streamed AI Courses* | *5.988* | *0.914* |
